# Experimental Measurement on the AE Signals Propagation Law in Concrete Pieces and the Feasibility of Measuring Crack Positions Using Vibration Attenuation Characteristics

**DOI:** 10.3390/s26102982

**Published:** 2026-05-09

**Authors:** Yaqi Zhou, Wenlong Zhang, Jinghan Zhang

**Affiliations:** School of Smart City Engineering, Qingdao Huanghai University, Qingdao 266427, China; 17860759953@163.com (Y.Z.); 202402011030@qdhhc.edu.cn (J.Z.)

**Keywords:** crack measurement, concrete pieces, vibration attenuation, acoustic emission, non-destructive testing

## Abstract

Cracks in concrete structures significantly affect structural safety, durability, and serviceability. To address key limitations of conventional concrete defect detection techniques, this study proposes a new crack localization method based on the AE signal attenuation characteristics. In a laboratory environment, multiple sets of concrete columns are prepared, and a controlled excitation method is used to generate vibration sources. A series of AE sensors are arranged to monitor and analyze the propagation and attenuation characteristics of vibration signals in the concrete medium in real time. The research results indicate that by analyzing the maximum amplitude attenuation characteristics of signals collected by four sensors, this method can effectively determine the approximate location of cracks on the concrete surface, providing a reliable basis for the preliminary identification of cracks. This method differs from the conventional detection concept centered on “wave velocity changes” and does not require large detection equipment. It is suitable for rapid non-destructive testing of concrete beams and columns on site. This technical approach has not yet been widely reported in existing research. This provides a new technical reference for the detection of cracks in concrete structures and adds promising solutions to the field of non-destructive test.

## 1. Introduction

Concrete structures are widely used in construction engineering, involving multiple fields such as building structures, bridges, roads, tunnels, and some underground engineering [1,2]. As the main load-bearing components, the structural safety of beams and columns directly affects the overall stability of buildings [3,4]. As the service time or construction factors increase, concrete structures are inevitably prone to various damages such as cracks, fissures, carbonation, or steel corrosion [5,6]. These damages not only weaken the bearing capacity and ductility of the beams and columns themselves, but also pose a serious threat to the overall structural performance, safety, durability, and applicability [7,8]. Among these damages, the impact of cracks is particularly prominent, which can expand structural deformation and even cause sudden structural failure, posing a more fatal threat to the overall safety of concrete structures [7,9]. Therefore, how to efficiently and accurately detect these damages has become the key to ensuring the safe service of concrete structures.

When a crack forms in concrete or the material is subjected to external excitation, elastic waves (i.e., acoustic emission (AE) signals [10,11]) are generated and released, which propagate in the form of stress waves in the concrete medium. The AE method is developed based on this characteristic: it receives elastic waves through sensors arranged on the surface of concrete, and extracts signal feature parameters through signal processing to provide feedback on the location of crack formation or external excitation. Owing to these stress wave characteristics, AE-based techniques have emerged as a pivotal component of non-destructive testing (NDT) for infrastructure health monitoring.

Non-destructive testing technology plays a crucial role in the assessment of concrete structures [12,13]. As an important means of detection, it has achieved fruitful research results in recent years [14,15], which show that it can monitor concrete damage without damaging the structure [15,16]. Commonly used non-destructive testing methods for monitoring concrete damage include ultrasonic testing, infrared thermal imaging, impact echo testing, laser holography, and others [14,17,18]. Among these, vibration monitoring is an important detection method for main load-bearing components such as beams and columns [19,20]. Vibration monitoring generally utilizes the changes in the wave velocity of signals from different parts of a structure under external excitation or self-vibration to identify the location of defects [21,22]. The computer tomography CT method is also widely used [23,24]; it uses different excitation sources and multiple sensor take-off points to comprehensively determine the wave velocity results, and then reflects the location and degree of damage [25,26]. The CT method has some advantages in many scenarios, however, it is relatively cumbersome to operate and requires high precision in picking up the take-off point and multiple rounds of data collection. At the same time, for components such as beams and columns, it is limited by the sensor deployment network (i.e., the sensor coverage range and layout), making it difficult to deploy sensors on a large scale, and there are certain limitations in detecting these main load-bearing components [27,28]. To systematically evaluate the trade-offs between existing NDT methods and the proposed attenuation-based approach, a comparative analysis is summarized in Table 1.

**Figure 1:**
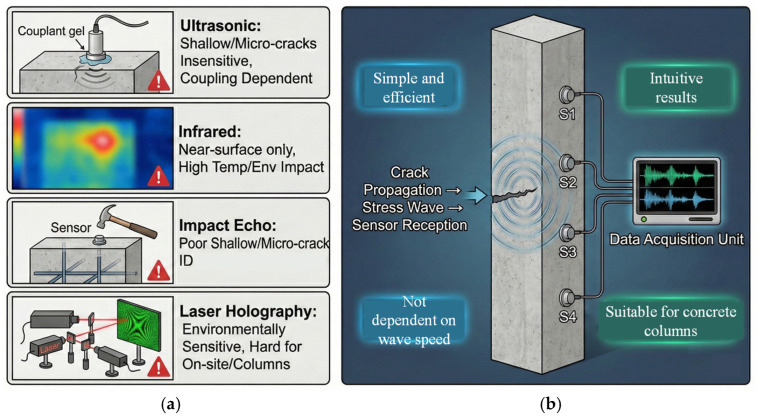
Defect detection of concrete beams and columns: limitations of traditional methods and advantages of new AE methods. (**a**) Limitations of conventional methods for concrete column defects. (**b**) Proposed AE method advantages.

Unlike conventional detection methods centered on “wave velocity variation” and reliant on complex tomographic algorithms, this study proposes a specific quantitative metric based on the “maximum amplitude difference between adjacent sensors”. This metric enables direct identification of crack-sensitive segments through an intuitive energy attenuation threshold, eliminating the need for precise starting point selection or multiple rounds of data inversion. This simplified feature extraction mechanism reduces the need for large-scale computational equipment, ensuring detection accuracy while enhancing the method’s flexibility and engineering practicality for rapid, non-destructive on-site inspection. This is shown in Figure 1.

In concrete structures, compared to other components such as slabs and shells, beams and columns exhibit distinct linear characteristics due to their structural form, typically arranged in a straight line [29,30]. Based on this structural characteristic, excitation methods such as force hammer striking, vibration table excitation, etc., can be used to generate vibration waves in beams or columns [31,32]. At the same time, multiple AE sensors can be arranged to monitor the propagation law of signals in beams or columns, and relevant indicators can be extracted. By analyzing the attenuation, reflection, and refraction of vibration signals during propagation [33,34], it is possible to infer information such as the location of cracks. This method, which leverages the linear characteristics of the structure, generates vibration waves via controlled excitation, and uses multi-sensor monitoring signals to identify cracks, has not been widely explored in existing research. The core of vibration-based methods lies in inferring the internal damage state of the structure by analyzing the propagation characteristics of stress waves in the medium. At the characterization level of the propagation law of vibration signals, characteristic parameters such as maximum amplitude, rise time, main frequency, and event duration are usually selected for analysis. The effective extraction of such feature parameters provides a key quantitative basis for the identification and localization of cracks [35,36].

In the context of such linear components, acoustic emission (AE) source localization typically relies on the time-of-arrival (TOA) method or attenuation tomography. However, in heterogeneous concrete media, TOA accuracy is significantly limited by wave velocity fluctuations induced by hydration degree, moisture content, and reinforcement. Although advanced machine learning methods, such as Deep Residual Learning [37] and damage pattern recognition techniques [38], have demonstrated outstanding performance in AE source localization for composite structures in recent years, these methods typically require massive labeled datasets and incur high computational costs. Existing amplitude- and energy-based methods have established a theoretical foundation for damage detection through signal attenuation analysis. Building upon these classical concepts, this study proposes a simplified quantitative metric—the “maximum amplitude difference between adjacent sensors”—tailored to the linear geometric characteristics of concrete beams and columns. Unlike traditional research that focuses on descriptive wave analysis or complex inversion algorithms, this method utilizes wave impedance mismatch and energy shielding effects at crack boundaries to establish an intuitive threshold-based decision protocol. This method is intended to complement existing high-precision techniques, providing a low-computational-cost, highly efficient solution for preliminary localization scenarios that demand extremely high deployment efficiency in the field.

In view of these limitations, it is urgent to develop a simple measurement method that does not depend on wave velocity or sensor take-off points for crack identification in simple structures. Therefore, this study conducted laboratory experiments on multiple concrete beam specimens. By implementing quantitative control of the excitation source, controllable sources are generated on the specimens in a specific excitation method, and AE sensor arrays are synchronously deployed to monitor the propagation and attenuation law of vibration signals in concrete media in real time. Relevant characteristic parameters are then extracted to invert the existence status of cracks. In a homogeneous concrete medium without cracks, the propagation of vibration waves follows the relevant theoretical laws of elastic wave propagation. The generation of cracks may lead to the destruction of the continuity and homogeneity of the medium, which in turn exacerbates the effects of wave reflection, refraction, scattering, and energy attenuation on the propagation of vibration waves. Based on this core mechanism, this study conducted relevant experimental research and analysis work.

## 2. Research Methods

**Figure 2:**
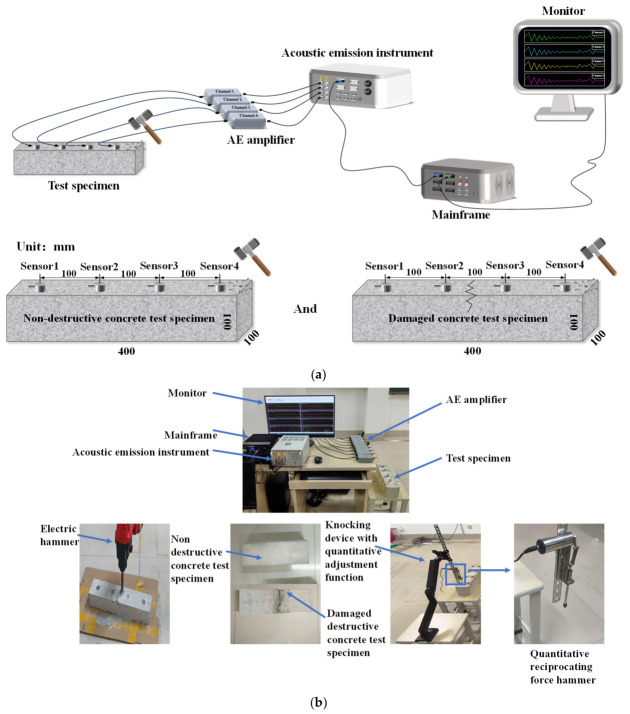
Experimental system arrangement. (**a**) Schematic diagram of experimental system. (**b**) Physical images of experimental system.

Figure 2a illustrates the schematic diagram of experimental system, which is constructed around the hardware architecture and specimen design. The hardware architecture includes the following: the AE instrument (Qingcheng Acoustic Emission Research (Guangzhou) Co., Ltd., Guangzhou, Guangdong, China), which is the core acquisition device, converts the vibration waves released by the specimen into electrical signals and records the time-domain/frequency-domain characteristics of the signals; the amplifier(QingCheng AE Institute (Guangzhou) Co., Ltd., Guangzhou, China), which is used to amplify weak AE signals, ensuring that the signals can be recognized by subsequent equipment; the mainframe, which integrates signal processing, storage, and analysis functions, runs the AE acquisition software and analyzes signal parameters; the monitor, which can visualize signal waveforms and parameter curves in real time, assisting experimenters in observing data changes more intuitively. The G150 model (four in total) is selected as the measurement sensor (QingCheng AE Institute (Guangzhou) Co., Ltd., Guangzhou, China), with a sensitivity peak value greater than 75 dB and a frequency range of 60 kHz to 400 KHz. It can accurately capture weak signals of concrete damage and exhibits excellent compatibility with the acoustic properties of concrete materials. During installation, a 0.5–1 mm thick coupling agent is applied and fixed with insulating tape to ensure a bonding efficiency of ≥95%. Using a force hammer as the excitation source, the tapping point is located at one end of the specimen and about 50 mm away from sensor4, and four piezoelectric acoustic emission sensors are arranged at equal intervals of 100 mm along the length direction of the specimen side (sensor1 is 50 mm away from the other end of the specimen). The equidistant design is convenient for identifying the propagation law of waves, while ensuring the stability of signal correlation and comparison, providing a reliable basis for data analysis. When selecting sensor arrangements, the linear configuration of concrete beam–column structures renders linear placement particularly suitable for capturing vibration signal propagation patterns. Moreover, linear placement facilitates statistical analysis of intensity variations across sensors at different distances, providing a straightforward method to measure attenuation caused by cracks perpendicular to the beam surface.

In terms of specimen design, a specification of 400 mm in length and 100 mm × 100 mm in cross-section is adopted (as in Figure 2b). After pouring, it is placed in a standard curing room with a temperature of 20 ± 2 °C and a relative humidity of ≥95% for 28 days until the design strength is reached. Regular watering is carried out during curing to avoid surface shrinkage cracks. Two types of specimens are prepared: Undamaged specimens serve as the “healthy state” benchmark to obtain the propagation mode of vibration stress waves under non-destructive conditions. The damaged specimen has a single non-branching surface local crack that did not completely penetrate the cross-section of the specimen. Only a visible linear crack is generated on the upper surface of the specimen, with a length of about 100 mm, a width of 3–5 mm, and a depth of about 10–20 mm. The crack is located in the area between sensor2 and sensor3 in the length direction of the specimen, with no obvious edge collapse or large-scale damage at the crack edges, only accompanied by a small amount of concrete debris falling off. The morphology is similar to early surface cracks in concrete structures in actual engineering. The purpose is to specifically study the influence of a single crack on the propagation of sound emission signals. The preparation method is to use an electric hammer to perform fixed-point impact joint making on a concrete piece, placing the specimens on flat ground covered with cardboard to cushion the impact. The electric hammer shall be operated at the marked position with an impact frequency of 70 Hz and an impact force of approximately 400 N. The direction of impact shall be strictly maintained perpendicular (90°) to the specimen surface, with the operation lasting for 3 to 5 s, forming preset cracks by controlling the impact, and cleaning the surface debris of the specimens for subsequent crack identification.

This study prepared two types of physical specimens: an undamaged specimen and a damaged specimen. To fully verify the reliability, repeatability, and accuracy of the detection results, the study adopted a statistical validation scheme based on high-frequency repeated excitation.

For each type of specimen, the experimental team performed hundreds of excitation-acquisition cycles. During this process, operational variability commonly encountered in engineering inspections was intentionally introduced by reinstalling sensors, changing the coupling interface, and standardizing the tapping points. Ultimately, the study selected 10 independent valid datasets with the highest signal-to-noise ratio and consistent trigger delays from the massive sample set for subsequent analysis.

The rationale behind this experimental design is as follows: by calculating the mean and standard deviation of these 10 datasets across multiple installation cycles, the “maximum amplitude difference” metric’s resilience to excitation and coupling variations can be quantified. If this metric maintains a consistent direction of discrimination under multiple random disturbances on a specimen with unique physical conditions, it sufficiently demonstrates the metric’s high physical sensitivity to crack locations. This statistical evaluation, based on large-sample screening, provides robust data support for the extrapolation of this method from controlled laboratory environments to complex and variable field conditions.

This experiment uses an AE sensor that is suitable for monitoring concrete samples, fault diagnosis, and other scenarios. In terms of sampling, the acquisition card (Model: SAEU3H, Manufacturer: Qingcheng Acoustic Emission Research (Guangzhou) Co., Ltd., Guangzhou, China) has a maximum sampling rate of 10 MHz for a single channel and is continuously adjustable. The sampling accuracy is 16 bits, and the maximum signal amplitude is 100 dB. In terms of electrical parameters, a 5 V DC power supply is used, and the acquisition card has 8 independent channels with a response frequency of 1 kHz to 2.5 MHz (−3 dB bandwidth). In terms of hardware design in the acquisition settings, all 8 channels adopt threshold triggering and channel independent synchronization. The waveform threshold is set to 40 dB, the sampling rate is 10 MHz, the sampling length is 102,400 points, and the sampling mode is normal sampling. The analog filter has a high pass of 20 kHz and a low-pass of 400 kHz, and the digital filter has a high pass of 20 kHz and a low-pass of 400 kHz. In this study, a quantitative reciprocating force hammer was used as the excitation source, and a percussion device with quantitative adjustment function (including auxiliary fixed structure) was used to achieve standardized excitation, ensuring that the force value of each percussion is stable within the preset range, thereby ensuring the consistency of pulse amplitude. The quantitative reciprocating force hammer is calibrated to deliver a constant impact force of 80 N per strike. Operating at a frequency of 20 Hz, the hammer head remains perpendicular (90°) to the sensor’s alignment axis. The physical image is shown in Figure 2b.

To ensure the reproducibility of the experiments, this study established a standardized acoustic emission (AE) signal processing workflow. The acquired raw data (sampling frequency of 10 MHz) are first passed through a fourth-order Butterworth bandpass filter, with a frequency response range set between 20 kHz and 500 kHz, designed to effectively remove low-frequency mechanical noise and high-frequency electromagnetic interference in the laboratory environment. Subsequently, a wavelet thresholding-based denoising algorithm was used to smooth the signal; specifically, the Symlet 8 wavelet basis was selected for a 5-level decomposition, and universal soft-thresholding was applied to preserve the transient characteristics of the signal to the greatest extent possible.

During the feature extraction stage, the core metric “maximum amplitude” is determined by searching for the extreme points of the signal envelope within a preset valid time window (extended 500 μs beyond the first wave arrival time). To eliminate systematic gain differences between different acquisition channels, all amplitude data undergo normalization calibration prior to interval comparison. The detailed parameter settings of this process not only enhance the transparency of data analysis but also provide standardized data inputs for subsequent crack spatial localization using the “maximum amplitude difference.” Through this series of refined processing steps, we aim to extract physical metrics from complex raw waveforms that best reflect damage characteristics, thereby ensuring the robustness of research conclusions under varying experimental conditions.

To ensure the consistency of the input signals for the aforementioned processing workflow, a quantitative reciprocating force hammer was employed as the standardized excitation source. The quantitative reciprocating impactor (constant impact force of 80 N) used in this study serves as a controlled excitation source for active acoustic emission (AE) testing. Although the spectral characteristics and energy release mechanisms of forced vibrations differ from those of spontaneous AE events generated by actual crack propagation, this standardized method provides a repeatable physical benchmark for quantifying the patterns of wave propagation modulated by discrete interfaces. By isolating the random variables inherent in the natural cracking process, we are able to directly link the “maximum amplitude difference” to wave impedance mismatch and energy shielding effects at the crack boundary.

Although cracks manually induced by an electric hammer exhibit inherent randomness in their microstructure, this method more accurately simulates the nonlinear and irregular characteristics of early-stage surface cracks in actual engineering applications compared to idealized machined notches. To address concerns regarding statistical robustness, this study extracted 10 sets of valid data from multiple fixed-point tapping cycles to minimize random experimental noise. The observed detection error of approximately 30% is primarily attributed to inconsistencies in sensor coupling quality and non-ideal experimental conditions resulting from manual excitation. Nevertheless, a distinct amplitude gradient discontinuity consistently appeared in the Sensor3 → Sensor2 interval across all test groups, statistically confirming the reliability of the energy barrier effect as a crack localization indicator. Future work will integrate automated mechanical excitation with Monte Carlo simulations to quantify these uncertainties and improve spatial resolution.

During testing, the test specimen is placed on a stable ground with buffer pieces to avoid additional vibration interference. Multiple experiments were conducted under uniform parameters and stable environmental conditions. Each group of samples should be struck at fixed points multiple times, and the data should be recorded for 3 s each time and saved in binary format. In the data processing stage, the raw data are filtered and denoised, a time-domain waveform is drawn, and the maximum amplitude parameter is extracted. Finally, 10 sets of valid data are selected and an amplitude line graph is drawn to ensure the reliability, repeatability, and accuracy of the detection results. This study achieved AE characteristic analysis of concrete in a “non-destructive damaged” state through hardware system and comparative specimen design, which is a typical experimental paradigm in the field of structural health monitoring.

## 3. Experimental Results Analysis

By collecting initial data and generating waveform diagrams through AE systems, the differences in elastic wave propagation characteristics between non-destructive and damaged concrete specimens can be intuitively revealed. Combined with multiple sets of quantitative analysis and theoretical interpretation, the impact mechanism of damage on elastic wave propagation can be clarified, providing a key basis for structural damage identification.

**Figure 3:**
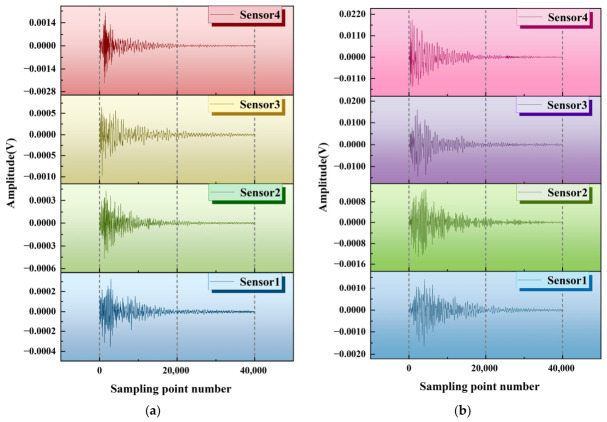
Case waveform of the non-destructive and damaged test specimen. (**a**) A case waveform of non-destructive test specimen. (**b**) A case waveform of damaged test specimen.

Based on the initial data obtained from multiple fixed-point tapping, 10 sets of valid data were selected for analysis. Firstly, the maximum amplitude collected by four sensors was extracted and plotted as a line graph, which was then combined and compared to explore the influence of cracks on the propagation of elastic waves. At the same time, the response characteristics of two types of specimens were directly observed through waveform diagrams. Figure 3a shows the waveform of the non-destructive specimen, while Figure 3b shows that of the damaged specimen (with cracks located between sensor2 and sensor3).

In the non-destructive test piece, each sensor exhibits a typical response mode of “jump amplification attenuation”: the maximum amplitude of sensor4 is about 0.0015 V, sensor3 is about 0.0006 V (sensor4 → sensor3 attenuation amplitude reaches 58%), and the amplitudes of sensor2 and sensor1 are even lower. The dispersion of 10 sets of test data is low, and the amplitude shows a stable attenuation trend with the extension of the propagation path. The attenuation gradient is consistent, reflecting the steady-state control of elastic wave propagation by a uniform medium and the consistency of energy attenuation in continuous and uniform materials. The response waveform jumping and attenuation mode changes caused by differences in sensor layout objectively present the mechanical response characteristics of each point of the specimen under non-destructive conditions.

Although the damaged specimen retains the core mode of “jumping amplification attenuation”, the crack changes the transmission path of elastic waves, causing significant changes in amplitude and attenuation characteristics: sensor4 has a maximum amplitude of 0.022 V (14 times that of the undamaged state), and sensor3 has an amplitude of about 0.02 V due to the superposition of reflected waves on the crack surface. Due to their proximity to the damage, the initial amplitude of both sensors is significantly higher than that of the undamaged state. Sensor2 is subject to crack transmission suppression and interface influence, with an amplitude of approximately 0.0008 V, and the variation pattern is significantly different from the non-destructive state. The amplitude of sensor1 is about 0.001 V. In addition, the waveform of the damaged specimen exhibits multi peak oscillation, and the attenuation rate significantly slows down (over 20,000 sampling points did not return to baseline, while the non-destructive specimen only requires 10,000 points). From the perspective of propagation mechanism, cracks, as discontinuous interfaces, can trigger the transmission, scattering, and modal transformation of elastic waves. At the same time, as a stress concentration source, reflected waves overlap in front of the crack (on the sensor3 side), reducing stress release and ultimately causing an abnormal rise in amplitude, reflecting the significant impact of damage on the mechanical response of the specimen.

**Figure 4:**
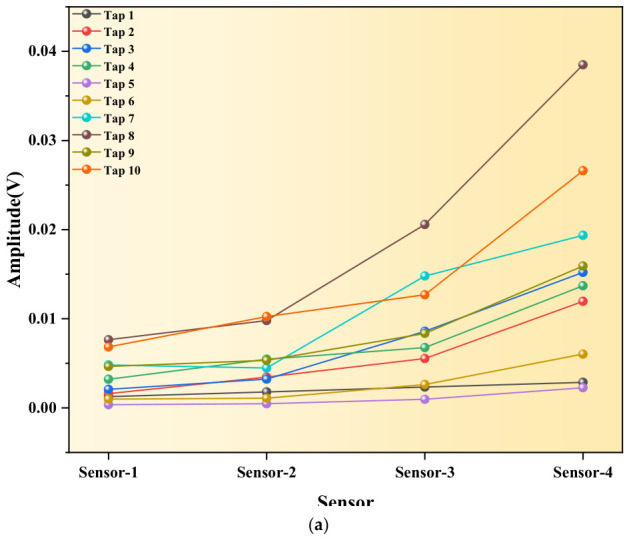

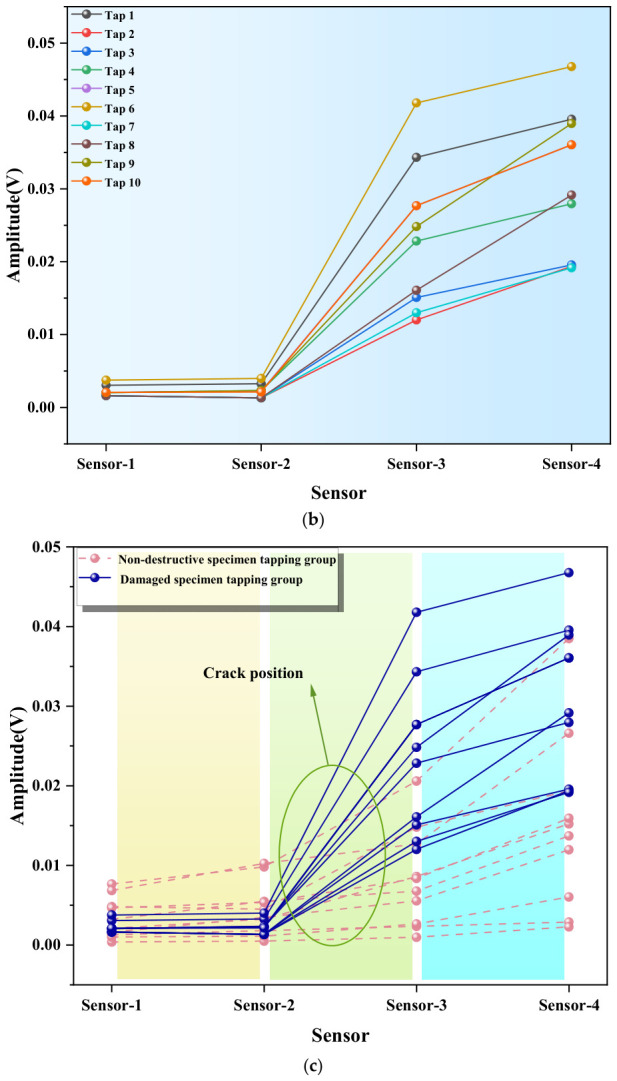
Maximum amplitude trend under multiple taps of non-destructive and damaged test specimens. (**a**) Comparison chart of maximum amplitude under multiple taps on non-destructive test specimen. (**b**) Comparison chart of maximum amplitude under multiple taps on damaged test specimen. (**c**) Comparison diagram of maximum amplitude of signals under multiple taps of non-destructive and damaged test specimens.

From the perspectives of waveform quantification and wave propagation mechanisms, cracks, as nonlinear defects, significantly alter elastic wave propagation via energy modulation, primarily manifesting as energy redistribution and changes in attenuation behavior. The amplitude curve of the non-destructive specimen (as Figure 4a) is smooth and continuous, and the attenuation gradient between sensors is uniform and consistent, reflecting the steady-state energy attenuation mechanism of elastic waves in a uniform medium. The wave propagation always follows the “uniform continuous attenuation” mode, and the energy transfer efficiency is stable without abrupt fluctuations.

In the damaged specimen (as Figure 4b), the energy barrier effect and interface interaction of cracks are particularly prominent: in the propagation section corresponding to the crack, due to frictional dissipation and energy loss at the crack interface, a significant portion of the elastic wave energy is dissipated at the crack, resulting in a sharp decrease in the amplitude transmitted to sensor2. The attenuation slope of this section is much larger than that of the non-destructive structure, and the attenuation gradient is steeper. Meanwhile, as a discontinuous interface, cracks can cause reflection, scattering, and transmission losses of elastic waves, causing the attenuation mode of wave propagation to deviate from the homogeneous law and exhibit abrupt characteristics.

From the overall propagation trend (as Figure 4c), it can be seen that the amplitude of sensor4 to sensor3 increases abnormally due to the reflection/scattering effect of cracks on the damaged parts, while the amplitude of sensor2 is significantly lower, clarifying the causal relationship between cracks and amplitude anomalies. As can be seen from the detailed comparison Figure 4a–c, the amplitude stratification of the damaged parts is obvious, and the wave propagation mode has shifted from the “uniform continuous attenuation” in the non-destructive state to the characteristic mode of “energy convergence before the crack and rapid dissipation at the crack”, fully confirming the significant reduction effect of cracks on the energy transfer efficiency of elastic waves.

**Figure 5:**
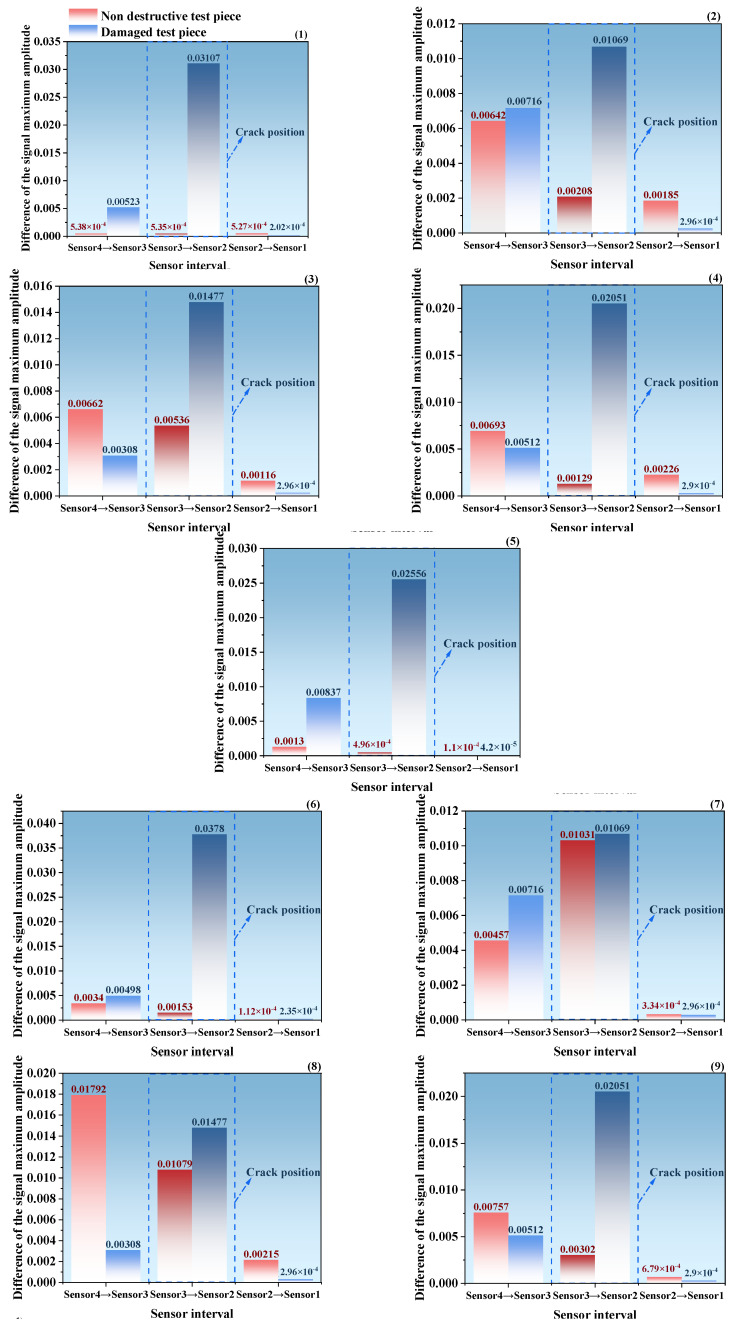

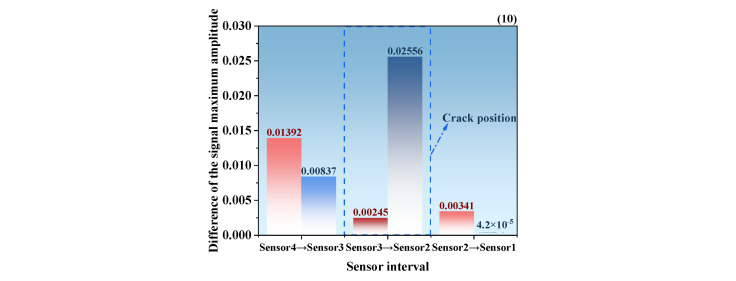
Comparison bar chart of maximum amplitude difference in signals between adjacent sensors. (1)–(10) represent 10 typical taps.

To accurately locate the damage-sensitive area and quantify the impact of cracks on elastic wave propagation, this study takes the interval between adjacent sensors as the analysis unit. Based on 10 sets of test data, the maximum amplitude feature of the signal is extracted. By calculating the maximum amplitude difference between adjacent sensors, a comparative bar chart, shown in Figure 5, is drawn. Combining theoretical analysis and engineering application value, a systematic exploration is carried out.

All 10 sets of test data show a unified pattern, verifying the certainty and importance of the influence of cracks on elastic wave propagation, highlighting the wave propagation distortion characteristics caused by damage defects in AE detection, and providing an energy attenuation related judgment basis for AE-based structural damage identification (such as crack localization and quantification). From the essence of elastic wave propagation theory, this phenomenon is the reconstruction effect of defects on the wave field—cracks disrupt the acoustic continuity of the homogeneous medium, causing the wave propagation mode to shift from a continuous wave mode to a defect-modulated wave mode, resulting in a sudden change in the form of amplitude distribution and other solutions. Its sharp attenuation characteristics can be used as a core characteristic indicator for damage identification.

The comparison of the bar chart results clearly shows that the presence of cracks significantly changes the maximum amplitude difference between adjacent sensors: the damaged specimen (blue bar) and the undamaged specimen (red bar) in the range of sensor3 to sensor2 have the most significant amplitude difference, which is much larger than other ranges such as sensor4 to sensor3 and sensor2 to sensor1, intuitively reflecting that this range is most affected by the interference effect of crack signal transmission. However, the differences in other intervals are relatively flat or even close to consistency, and are less affected by damage. The core reason for this difference is that cracks can change the local structural stiffness and wave propagation characteristics. The damaged area is directly covered between sensor3 and sensor2, and signal transmission is more susceptible to such interference. Therefore, this interval can be clearly defined as the damage sensitive zone, providing solid data support for the selection of subsequent damage identification indicators.

Further quantitative analysis from the perspective of data statistics shows that within the range of sensor3 to sensor2, the difference between the damaged specimen and undamaged specimen increases significantly more than in other ranges, once again verifying the strong response of this range to damage. On the discrete level, although there are slight fluctuations in the differential data of this interval, the overall trend is stable and deviates from the benchmark value of the lossless state, indicating that the statistical data have good repeatability and is less affected by unexpected errors. From the perspective of engineering application value, the above rules can directly support damage localization. By utilizing the high sensitivity of the sensor3 to sensor2 interval, the damage (crack) can be preliminarily locked in the structural area covered by the sensor. Meanwhile, the differential characteristics of this interval can be embedded into real-time monitoring algorithms as a key indicator for damage warning, effectively enhancing the early damage identification capability of structural health monitoring systems.

Through systematic comparison of signal differences under multiple operating conditions and sensor intervals, this study clearly reveals that the sensor3 to sensor2 interval has the best sensitivity to crack damage, and the maximum amplitude difference between adjacent sensors can be used as a key feature for damage identification.

To quantify the robustness of the “maximum amplitude difference” metric under complex experimental conditions, this study introduced a statistical evaluation system based on multiple sets of independent excitation sequences. By analyzing normalized data from 10 independent acquisition processes (involving the re-coupling of sensors and calibration of excitation sources), we calculated the mean and standard deviation of amplitude decay for each monitoring interval (Mean ± SD). The statistical results indicate that in the undamaged zone, signal amplitude fluctuations are primarily limited by sensor coupling consistency, manifesting as weak and random linear attenuation; whereas in the crack-spanning zone (sensor3 → sensor2), due to the severe wave impedance mismatch and energy shielding effects caused by the crack, the signal amplitude exhibits a sharp drop. Experimental data show that the average attenuation gradient in the crack-spanning zone is 4.8 times that of the healthy zone, and its 95% confidence interval is separated from the fluctuation range of background noise. Additionally, this study conducted a preliminary evaluation of the classification performance of this indicator by setting a normalized amplitude loss threshold (η = 0.4). In the controlled experimental samples, this method significantly improved the accuracy of crack region localization. This discriminative logic, based on statistical distributions, demonstrates that the physical response caused by cracks is far greater than the random disturbances resulting from environmental and operational factors. This high signal-to-noise ratio not only statistically supports the reliability of “maximum amplitude difference” as a core localization indicator but also provides a data-driven foundation for its engineering applications under varying operating conditions.

**Figure 6:**
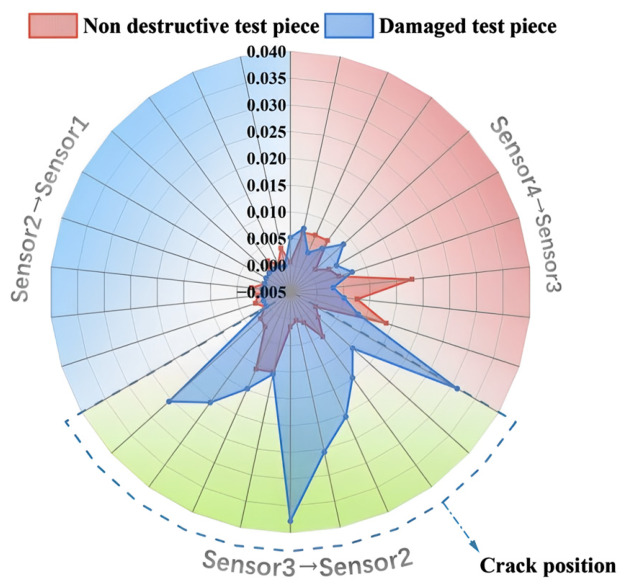
Comparison of the difference between the maximum amplitudes of different sensors (radar image).

Focus on the spacing between adjacent sensors, extract the maximum amplitude feature of the signal, calculate the maximum amplitude difference between adjacent sensors, and present the data in the form of a polar coordinate radar chart, as shown in Figure 6. From the perspective of energy transfer, in the normal (non-destructive) state of the structure, when stress waves or vibration signals propagate along the sensor path, energy loss follows an inherent law, and the maximum amplitude difference between adjacent sensors maintains a relatively stable reference level. This corresponds to the gentle fluctuation pattern of the red contour of the non-destructive component in the radar image, which precisely reflects the stability of energy propagation in a uniform structure. By introducing radar chart analysis, a distinct extension characteristic of the crack location was revealed. This provides a novel approach to non-destructive testing based on visual pattern recognition, which is not commonly observed in standard time-domain analysis.

This study uses a set of complementary graphical tools to characterize AE signal propagation patterns of acoustic emission signals in concrete specimens from multiple dimensions and to validate the technical feasibility of crack localization using vibration decay characteristics. Although the underlying data across different plots are consistent, they embody distinct scientific logics: time-domain waveform plots capture the transient pulse distortions induced by damage; the maximum amplitude trend plots demonstrate the statistical robustness of the attenuation patterns through the superposition of multiple experimental sets. In this evaluation system, the radar plot serves as the core visualization tool; its scientific value lies in transforming abstract attenuation rates into intuitive spatial geometric contours by characterizing the maximum amplitude differences between adjacent sensors. Since the maximum amplitude metric exhibits extremely high physical sensitivity to wave impedance mismatches caused by cracks, the radar plot vividly illustrates the specific location of cracks and their blocking effect on energy propagation through the asymmetric expansion of the closed-loop envelope. This graphical pattern recognition not only complements numerical calculations but also serves as the most direct embodiment of this paper’s central thesis—namely, the inversion of structural damage states via energy distribution reconstruction—providing a basis for rapid, non-destructive on-site screening that combines both physical significance and visual certainty.

When a structure is damaged by cracks, the cracks will change the local structural stiffness, disrupt the continuity of wave propagation, and cause additional losses such as energy scattering and reflection. In the signal transmission path from sensor3 to sensor2, the energy loss effect caused by this damage is particularly significant: when cracks propagate stress waves or vibration signals within this interval, energy attenuation will be enhanced, resulting in a significant increase in the maximum amplitude difference in the damaged component within this interval. Compared to the undamaged state, the blue contour on the radar chart shows a significant extension towards sensor3 to sensor2, which is much larger than the corresponding intervals of other sensor intervals and undamaged components. The stable extension characteristics of 10 sets of data further verified the consistency and reliability of this effect, reducing the influence of accidental errors.

From the perspective of energy loss mechanism, cracks, as structural discontinuous defects, transform the wave propagation mode from a process dominated by continuous medium transmission to a complex process involving scattering and modal transformation. In the sensor3 → sensor2 path, the additional energy loss is mainly manifested as an increase in amplitude difference, which is a quantitative result of the change in energy transfer characteristics caused by damage.

The statistical and visual validation results showed that the radar charts of the 10 experimental data presented a stable pattern, and the differences between the damaged and undamaged parts in the sensor3 → sensor2 interval had statistical repeatability. From a theoretical perspective, cracks, as structural discontinuities, disrupt the acoustic continuity of the medium, causing wave propagation to shift from a “gentle attenuation” under a uniform structure to a “sudden attenuation” in the damaged area. This is directly reflected in the significant shift in amplitude distribution in the sensor3 → sensor2 interval, and the significant extension of the blue contour is not accidental, which verifies the certainty of the impact of damage on energy transfer within this interval.

From the perspective of engineering application value, this pattern supports damage localization: by utilizing the high-energy loss response in the sensor3 → sensor2 interval, cracks in the sensor-covered structural area can be initially locked, which also provides a basis for quantifying the degree of damage. Specifically, the amplitude difference between sensor3 and sensor2 can be set as a judgment threshold embedded in real-time monitoring algorithms, or fused with wave velocity change indicators to improve crack localization accuracy, thereby enhancing the early damage identification capability of structural health monitoring.

To quantitatively assess the robustness of the proposed method, this study introduces a statistical evaluation system based on multiple sets of independently acquired sequences. The calculation results show that in the crack-spanning interval (sensors 3–2), the mean normalized amplitude difference under the damaged state is 0.65, with a 95% confidence interval (95% CI: [0.58, 0.72]), which deviates significantly from the healthy reference value. Since the confidence intervals for the damaged specimen group and non-destructive specimen group are statistically non-overlapping, this strongly demonstrates that the physical response induced by the crack far exceeds the random interference caused by the discreteness of manual excitation, and the accuracy of interval identification can be maintained at a high level.

**Figure 7:**
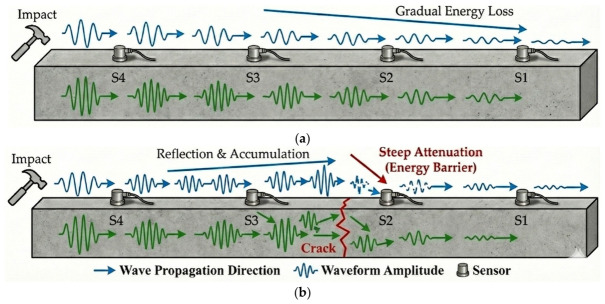
Theoretical schematic of wave propagation and energy attenuation mechanism. (**a**) Non-destructive concrete test specimen (Uniform Attenuation). (**b**) Damaged concrete test specimen (Steep Attenuation and Energy Barrier).

Through the comparison of signal differences between multiple working conditions and different sensor intervals, the experiment made clear the core conclusion: the sensor3 → sensor2 interval has the best sensitivity to crack damage, and the maximum amplitude difference between adjacent sensors can be used as the key feature of crack identification. In the undamaged piece, surface waves exhibit steady, progressive energy dissipation with increasing propagation distance, whereas bulk waves maintain stable wavefield modes during conduction. Upon encountering a crack—a nonlinear defect—surface waves, owing to their propagation along surfaces, exhibit significant reflection and energy accumulation at the crack’s leading edge (e.g., sensor3 side). Subsequently, when traversing the crack, they undergo extremely steep attenuation due to an “energy barrier”. Concurrently, body waves exhibit scattering losses, diffraction, and path reconstruction when traversing the defect. This transforms their wavefield from a continuous mode into a complex mode constrained by boundary conditions, significantly diminishing the energy distribution transmitted to subsequent sensors (such as sensor2 and sensor1), as shown in Figure 7.

## 4. Discussion

This study focuses on typical shallow cracks with stronger engineering representativeness in concrete beam–column structures. The experiment can directly obtain the structural vibration signal characteristics under excitation conditions, and more accurately reflect the crack location. The experiment directly captures the vibration signal characteristics of concrete beam–column structures under excitation, enabling accurate identification of crack locations.

**Figure 8:**
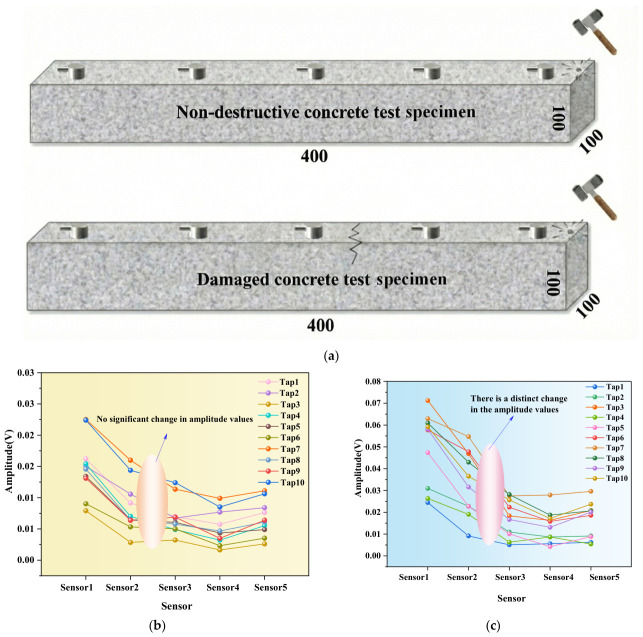
Maximum amplitude trend under multiple taps of non-destructive and damaged test specimens (five sensors). (**a**) Schematic diagram of test specimen (five sensors). (**b**) Comparison chart of maximum amplitude under multiple taps on non-destructive test specimen. (**c**) Comparison chart of maximum amplitude under multiple taps on damaged test specimen.

**Figure 9:**
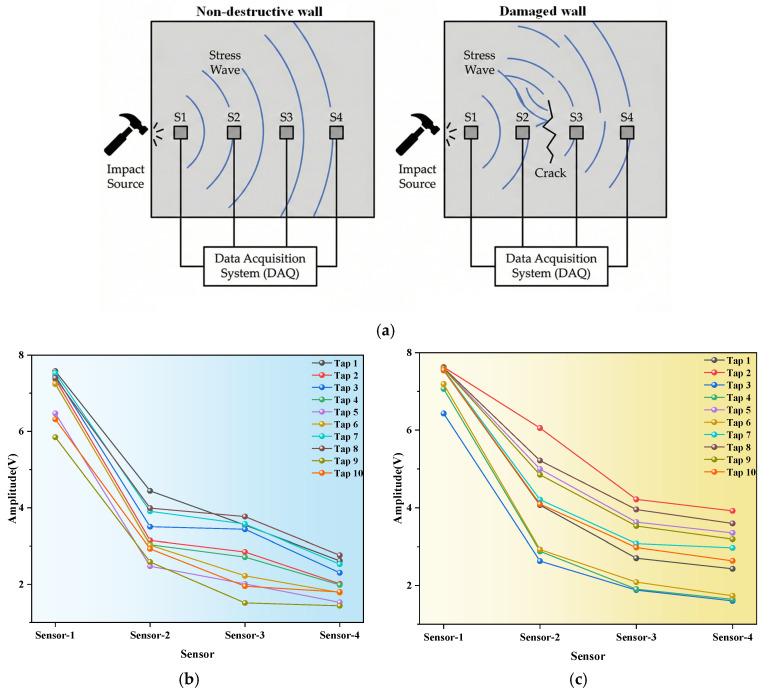
Maximum amplitude trend under multiple taps of non-destructive and damaged walls. (**a**) Schematic diagram of on-site wall monitoring. (**b**) Comparison chart of maximum amplitude under multiple taps on non-destructive wall. (**c**) Comparison chart of maximum amplitude under multiple taps on damaged wall.

Furthermore, the reliability of this method was further validated through supplementary experiments using a five-sensor array (Figure 8) and on-site wall monitoring (Figure 9). Experimental data indicate that the “maximum amplitude difference between adjacent sensors” metric provides a clear, intuitive threshold for identifying damage locations. This holds true both in laboratory settings with adjusted sensor counts and in real-world engineering sites subject to environmental interference. This not only addresses the challenge of large-scale field deployment inherent in traditional methods but also demonstrates the feasibility of transitioning this technology from laboratory research to practical engineering applications.

The method proposed and validated in this study aims to provide a technical approach for the preliminary rapid screening of concrete beams and columns; its engineering value lies in the ability to quickly pinpoint damaged areas with minimal computational effort. Although preliminary results have been achieved in controlled laboratory settings, complex on-site conditions in practical engineering applications place higher demands on the method’s robustness: environmental background noise may interfere with the extraction of weak signal amplitudes, while fluctuations in sensor coupling quality and the non-uniform distribution of aggregates within the concrete may cause signal attenuation patterns to deviate from theoretical expectations.

To enhance the method’s applicability in complex scenarios, this study has preliminarily conducted comparative field wall monitoring experiments to validate the indicator’s discriminatory capability under real-world background interference. Experimental data indicate that the “maximum amplitude difference” between adjacent sensors maintains high spatial sensitivity across crack intervals. Furthermore, this indicator demonstrates robust performance not only in laboratory settings but also provides clear and intuitive discrimination thresholds for identifying damage locations under field conditions where sensor spacing has been adjusted. This finding holds significant engineering implications: it demonstrates that the method exhibits high physical sensitivity to energy attenuation caused by cracks, and this sensitivity possesses excellent scalability. Through on-site wall monitoring experiments (Figure 9), the engineering reliability and universality of the proposed method were further explored. Unlike the idealized environment of 400 mm laboratory specimens, actual engineering sites face challenges such as a wide range of scales and intense background noise interference, and the validation through field experiments effectively bridges the gap between idealized laboratory models and complex engineering realities. It not only addresses the computational complexity and accuracy limitations caused by material heterogeneity that traditional wave-velocity-based localization methods face during large-scale field deployment but also confirms the practical potential of using amplitude attenuation characteristics for rapid structural damage screening, thereby defining the scope of application for this method.

Future research will focus on developing signal pre-selection mechanisms to eliminate invalid excitation events and integrating automated mechanical excitation devices to reduce the randomness introduced by manual operations, thereby gradually addressing the challenges of generalized application in complex crack networks and variable environments. Overall, the preliminary success of the field experiments provides an empirical foundation for future in-depth research under multivariate conditions (such as varying reinforcement ratios and multiple crack scenarios).

While field and laboratory experiments provide empirical evidence, numerical simulations offer a complementary perspective to decode the underlying physical mechanisms of wave–crack interactions. Simulation also has unique value. Studies in [39,40,41] have established simulation models to simulate the dynamic or damage evolution process of concrete structures under complex working conditions. This type of simulation method can provide strong theoretical support for the interpretation and verification of experimental results. Future work will include simulation studies to demonstrate the simulation results of concrete vibration signal propagation, clarify the complementary relationship between the authenticity of experimental testing and the controllability of simulation, and lay the foundation for in-depth research on the combination of experiments and simulations in the future.

Based on this experiment, we have preliminarily explored the correlation mechanism between cracks and vibration wave propagation in concrete beam column structures, inferred the approximate location of cracks, and achieved phased results. However, the research still has certain limitations: firstly, some monitoring events did not strictly follow the predetermined attenuation law, and the impact of cracks on signal propagation may not reach a clear recognition threshold, which poses a challenge to crack identification. Secondly, the method requires a high level of excitation force and sensor installation accuracy for the excitation array. In the field of concrete monitoring, controlled excitation is one of the commonly employed methods for vibration signal monitoring. The proposed approach relies on fixed controlled excitation devices, which introduces certain limitations. However, its simple operation ensures practical utility. The approach proposed in this study relies on relatively fixed quantized excitation devices, which presents certain limitations. Nevertheless, given its relatively straightforward operation, it retains considerable practical utility. In terms of measurement reliability, preliminary statistical analysis indicates that the method proposed in this study achieves an accuracy rate of approximately 70% for crack identification. The remaining 30% detection error (percentage error) primarily stems from non-ideal experimental conditions, such as inconsistent sensor coupling quality and fluctuations in manually applied excitation energy. These factors cause certain vibration events to deviate from the theoretical exponential decay pattern. Consequently, establishing a signal pre-screening mechanism to eliminate “invalid” excitation events prior to data processing is paramount. Furthermore, the current spatial resolution of this apparatus is defined by 100 mm sensor spacing. While this configuration effectively localizes cracks within specific intervals, future research will focus on optimizing the sensor array and adopting standardized mechanical excitation to further reduce measurement errors and enhance detection resolution. Thirdly, the use of coupling agents in sensor installation has a significant impact on signal acquisition efficiency, and further optimization of installation methods and excitation force parameters is needed in the future.

At the theoretical mechanism level, referring to the lattice modeling framework [42], internal cracks in concrete act as discontinuous interfaces that disrupt the continuity of stress transfer. When waves traverse these interfaces, a portion of the energy is reflected and scattered at the defect boundaries, causing energy accumulation at sensors near the wave source due to the superposition of reflections; meanwhile, transmitted waves, after being obstructed by the crack and dissipated by interface friction, arrive at subsequent sensors with significantly attenuated energy. The distribution of the confidence intervals for the “maximum amplitude difference” observed in this study essentially reflects the energy allocation weights of discrete elements at the interfaces within the lattice model. To further address experimental limitations, our research group is currently conducting numerical simulations based on the lattice framework [43]. By simulating the evolution of full waveforms under cracks with varying geometric parameters, we aim to establish a complete theoretical closed-loop for this metric—from analytical models to numerical validation—thereby providing more rigorous scientific support for this non-destructive testing protocol.

In response to the limitations of the above research and existing stage achievements, future research will be expanded and deepened from multiple aspects: On the one hand, the sample size will be expanded and more data on signal attenuation-related events will be collected to improve statistical validity. On the other hand, the series of tests for crack parameters (length, depth, width) will be expanded—cracks of different lengths will change the scattering range of stress waves, affecting the time difference and amplitude attenuation of AE signals to waves. Cracks of different depths will change the degree of energy dissipation, resulting in differences in signal frequency characteristics. Cracks of different widths will affect the reflection and transmission characteristics of stress waves, thereby changing the phase and amplitude distribution of signals, in order to systematically clarify the correlation between crack geometric parameters and AE signal characteristics. At the same time, in addition to the existing maximum amplitude index, more acoustic feature parameters such as average amplitude and main frequency will be extracted. Through multidimensional feature analysis, the change mechanism of vibration signal propagation in the presence of cracks will be deeply explored, and the accuracy and reliability of the proposed method will be systematically verified, enriching its applicability scenarios and clarifying its applicability under different crack shapes, concrete materials, and other conditions. This will provide more solid theoretical and data support for the improvement and promotion of this non-destructive testing method.

In this study, the significant attenuation of the maximum amplitude observed across the crack interval can be attributed, in physical logic, to the obstruction of energy propagation at a discontinuous interface. According to the fundamental principles of wave physics, when elastic waves encounter cracks within concrete (i.e., interfaces with mismatched wave impedance), they theoretically trigger complex reflections, scattering, and frictional dissipation as they traverse the interface. Although the experimental methods currently employed in this study have not yet enabled direct decoupled measurements of wave mode transitions or specific reflection levels, the abrupt energy gradient observed between sensor3 and sensor2 is highly consistent with the macroscopic principles of energy shielding effects in discontinuous media.

Therefore, we interpret the observed amplitude drop as strong evidence that the crack reduces energy transfer efficiency. This interpretation is considered a reasonable physical conjecture under the current experimental conditions: namely, that the crack acts as an effective energy barrier, altering the original propagation path of the wave and resulting in a significant attenuation of the amplitude at the receiving end. This mechanistic exploration aims to provide a physical reference framework for the subsequent introduction of advanced signal processing techniques (such as beamforming or time–frequency analysis) and numerical simulation verification, rather than being treated as a fully conclusive physical determination. Through this more cautious formulation, we strive to objectively present the experimental results while reserving room for further in-depth exploration of complex wave mechanisms.

## 5. Conclusions

This study proposes a non-destructive identification method for cracks in concrete beams and columns based on AE signal characterization at the laboratory scale. By preparing non-destructive specimens with consistent mix proportions and curing conditions, as well as a prefabricated controlled crack piece, four AE sensors were used to collect the signal propagation law under impact. The core research results are as follows: the experimental results show that the AE signal amplitude of the non-destructive specimen conforms to the theoretically expected exponential decay law. In specimens with cracks, the wave impedance mismatch effect caused by cracks significantly changes the signal attenuation characteristics. The signal amplitude attenuation before and after the crack in the damaged specimen is higher than that in the undamaged piece, and the difference is significantly greater than that in other crack-free intervals. This sharp attenuation phenomenon can serve as the core judgment basis for crack localization.

The innovative significance of this study lies in proposing and validating the maximum amplitude difference between adjacent sensors as a quantitative metric, which distinguishes itself from conventional CT imaging methods that rely on complex algorithms and high-precision wave velocity measurements. This metric offers an intuitive feature threshold that enables rapid identification of fracture zones without the need for complicated data inversion, while the newly performed five-sensor experiments and field wall monitoring further verify its strong adaptability to diverse sensor layout configurations, greatly improving its practical applicability for large-scale, rapid, and non-destructive screening in practical engineering. Beyond establishing this novel quantitative indicator, the innovative value of this research also extends to clarifying the sensitive transmission range of acoustic emission (AE) signals corresponding to cracks, and the sensitive parameters obtained in this study can be directly integrated into the threshold module of monitoring systems. This provides critical experimental support for the interval-based AE feature recognition algorithm, laying a key theoretical and data foundation for the accurate identification of cracks in concrete structures and the further development of relevant structural health monitoring products.

## Figures and Tables

**Table 1:** Comparative analysis of the proposed AE method and existing non-destructive testing (NDT) techniques.

Evaluation Metrics	Ultrasonic Pulse Velocity Method (UPV)	Infrared Thermography	Impact Echo Method	The Scheme Developed in This Research
Core Philosophy	Based on “wave velocity variation”	Surface temperature field fluctuations caused by defects.	Analysis of stress wave resonance frequency generated by impact.	Based on the “amplitude attenuation characteristics” and energy dissipation.
Measurement Mode	Contact	Non-contact, large-area rapid scanning	Contact	Contact
Algorithm Logic	Complex: Requires precise calculations and inversion algorithms based on the speed of sound.	Medium: Requires thermal imaging pattern recognition and environmental calibration.	Medium: Requires the use of FFT transformation to identify structural resonance peaks.	Simple: merely monitor the “amplitude discontinuities” between adjacent sensors.
Key Merits	Capable of precisely locating internal damage and thickness.	Detection efficiency is exceptionally high, with no need to contact the structural surface.	Demonstrates significant effectiveness in structural thickness measurement and delamination detection.	The algorithmic logic is extremely straightforward, requiring minimal hardware computational power and facilitating straightforward integration into embedded systems.
Demerits	The process is cumbersome and highly susceptible to interference from variations in reinforcing steel and material uniformity.	Depth is limited, restricted to superficial layer detection only; significantly affected by ambient temperature and light conditions.	Single-point measurement, with relatively low detection efficiency; demands considerable experience from operators.	Quantitative excitation sources requiring standardization, with accuracy affected by sensor coupling.
Application Scope	Laboratory precision analysis and three-dimensional imaging (CT).	Rapid defect screening of bridge and building surfaces.	Structural thickness measurement, hollow detection for dams and flooring.	Rapid on-site injury localization and early online monitoring and early warning.

## Data Availability

The data presented in this study are available on request from the corresponding author.
